# Quantitative analysis of red blood cell membrane phospholipids and modulation of cell-macrophage interactions using cyclodextrins

**DOI:** 10.1038/s41598-020-72176-3

**Published:** 2020-09-15

**Authors:** Amid Vahedi, Parnian Bigdelou, Amir M. Farnoud

**Affiliations:** 1 Department of Chemical and Biomolecular Engineering, Ohio University, 161 Stocker Center, Athens, OH 45701 USA; 2 Biomedical Engineering Program, Ohio University, Athens, OH 45701 USA

**Keywords:** Membrane lipids, Membrane structure and assembly, Lipidomics, Lipids

## Abstract

The plasma membrane of eukaryotic cells is asymmetric with respect to its phospholipid composition. Analysis of the lipid composition of the outer leaflet is important for understanding cell membrane biology in health and disease. Here, a method based on cyclodextrin-mediated lipid exchange to characterize the phospholipids in the outer leaflet of red blood cells (RBCs) is reported. Methyl-α-cyclodextrin, loaded with exogenous lipids, was used to extract phospholipids from the membrane outer leaflet, while delivering lipids to the cell to maintain cell membrane integrity. Thin layer chromatography and lipidomics demonstrated that the extracted lipids were from the membrane outer leaflet. Phosphatidylcholines (PC) and sphingomyelins (SM) were the most abundant phospholipids in the RBCs outer leaflet with PC 34:1 and SM 34:1 being the most abundant species. Fluorescence quenching confirmed the delivery of exogenous lipids to the cell outer leaflet. The developed lipid exchange method was then used to remove phosphatidylserine, a phagocyte recognition marker, from the outer leaflet of senescent RBCs. Senescent RBCs with reconstituted membranes were phagocytosed in significantly lower amounts compared to control cells, demonstrating the efficiency of the lipid exchange process and its application in modifying cell–cell interactions.

## Introduction

The mammalian cell membrane is an asymmetric structure, with distinct lipids localizing in each leaflet of the membrane bilayer. The lipid asymmetry is not limited to lipid headgroups, but also lipid chain length and saturation^1^. Sphingomyelins (SM) and phosphatidylcholines (PC) primarily reside in the membrane outer leaflet (facing the outside environment) while the inner leaflet (facing the inside of the cell) is known to mainly consist of aminophospholipids, phosphatidylserines (PS) and phosphatidylethanolamines (PE)^2–5^. This phospholipid asymmetry has important implications for cell function and is known to affect a variety of processes, including apoptosis, cell division, and blood coagulation^6–9^. The functional importance of lipid asymmetry has led to increasing interest in understanding the lipid composition of individual membrane leaflets, with special interest in the outer leaflet, which is the first cellular entity that comes into contact with other cells, as well as external molecules and materials.

A detailed characterization of the lipid composition of individual membrane leaflets is particularly important for red blood cells (RBCs). RBCs are the first cells in which membrane asymmetry was revealed and are particularly suitable for studies on membrane lipid composition given that they lack internal organelles and therefore the majority of their lipids can be attributed to the membrane^1, 10^. The importance of membrane asymmetry in RBCs goes beyond their suitability as a model. The composition of the RBC membrane outer leaflet is known to change in a variety of diseases, including leukemia, sickle cell disease, thalassemia, and even diabetes^11–16^. In addition, loss of membrane asymmetry generally signals the end of the lifespan of RBCs. In senescent RBCs, loss of membrane asymmetry results in PS translocation to the membrane outer leaflet^17, 18^. This allows for senescent cells to be recognized and phagocytosed by macrophages through the action of Tim-1, Tim-4, Stabilin-2, and CD300 receptors or through binding of bridging molecules that facilitate recognition by integrins or TAM receptors^19, 20^. While this process is important for ensuring RBC homeostasis in the body, it has negative implications for RBC storage ex vivo. RBCs lose their normal concentration of ATP when stored in blood banking conditions and this facilitates the loss of asymmetry^21^, making transfused RBCs more prone to clearance by macrophages^17, 22, 23^. Therefore, a detailed analysis of the lipid composition of each membrane leaflet in RBCs is not only important for better understanding the membrane biology of these cells, but also aids in evaluating their changes in diseased conditions and during ex vivo preservation.

While the asymmetry of phospholipids in membrane leaflets has been known since the 1970s, the methods to specifically analyze the outer leaflet lipid composition have remained the same. Advances in mass spectrometry have made it possible to provide detailed and reliable information, as recently shown for total phospholipid content of human RBCs^24^. However, analysis of the outer leaflet lipid composition still depends on the enzymatic degradation of phospholipids. This method was effectively used, in combination with freeze-etch electron microscopy, in the study of Verkleij et al. to characterize the asymmetric distribution of phospholipids in the membrane of human RBCs, revealing significant levels of SM and PC in the outer leaflet and PE and PS in the inner leaflet^2^. The same method, in combination with mass spectrometry, was recently used by Lorent et al., reporting that the outer leaflet is composed of SM, PC, and a small amount of PS, while the inner leaflet primarily consists of PE, PS, PC, and small levels of SM^5^. Despite its value, enzymatic degradation of phospholipids is also known to have certain shortcomings, including enzyme specificity to different lipids as well as the possibility of cell lysis during the reaction^25, 26^. In addition, enzymatic degradation of lipids is dependent on the compression state of the membrane. For example, it has been shown that the ability of phospholipases to hydrolyze membrane lipids depends on the membrane surface tension with enzymes exerting their hydrolyzing activity only at certain membrane surface tension values^26–28^.

Host–guest chemistry using cyclodextrins, when combined with mass spectrometry, could provide a simple method to analyze the lipids of the membrane outer leaflet. Cyclodextrins are a group of cyclic oligosaccharide molecules with a structure that resembles a wreath-shaped truncated cone. The interior cavity of cyclodextrins is extremely hydrophobic and the exterior is hydrophilic, therefore, cyclodextrins can form an inclusion complex with the hydrocarbon chains of lipid molecules^29^. Cyclodextrins have been used to manipulate the lipid contents of lipid bilayers primarily in vesicles^30–35^. These studies have shown that α-cyclodextrins can form inclusion complexes with phospholipids, but not with cholesterol, as their cavity is not large enough to host cholesterol^35^. When inclusion complexes of phospholipids and cyclodextrins are in the vicinity of lipid bilayers, exogenous lipids can be exchanged between the bilayer and the cyclodextrin^36, 37^. The pioneering studies of Li et al. used this method to replace lipids of the outer leaflet of different mammalian cells with exogenous phospholipids using methyl-α-cyclodextrin (MαCD)^4, 38^.

The current study is the first effort to use cyclodextrin-lipid host–guest chemistry for the analysis of the lipids of the RBC outer leaflet. This method, in combination with mass spectrometry, reveals the lipid content and breakdown of individual lipid species in the outer leaflet. This method also delivers lipids to the outer leaflet of RBCs while depleting outer leaflet lipids, which can find applications in understanding the role of lipids in cell function and cell–cell interactions. As an application, we show that this method can be used to reconstitute outer leaflet of senescent RBCs and significantly reduce their clearance by macrophages, which can have implications for extending the ex vivo storage life-span of RBCs.

## Results

### Optimization of the lipid exchange process

To extract phospholipids from the RBC outer leaflet, a process adapted from Li et al., based on cyclodextrin chemistry, was used^4^. It was important to prevent the loss of cell viability during the extraction process, as loss of viability and disruption of membrane integrity would ‘contaminate’ the extracted lipids with lipids from the inner leaflet. To this aim, release of hemoglobin from the RBCs (a.k.a. hemolysis) was used to monitor cell membrane disruption^2^. Hemoglobin has an absorption peak at 541 nm; thus, hemolysis of RBCs during lipid extraction process could be readily measured using UV–Visible spectroscopy to ensure that the membrane integrity remained intact during outer leaflet extraction.

Addition of cyclodextrin to RBCs resulted in immediate hemolysis. Therefore, the strategy of Li et al., based on loading cyclodextrin with exogenous lipids and exchanging them with the lipids of the outer leaflet was followed^4^. It was reasoned that this exchange process will result in the inclusion of outer leaflet lipids in MαCD, allowing for analysis by chromatography and mass spectrometry. Therefore, the parameters involved in the lipid exchange process (lipid headgroup, lipid concentration, MαCD concentration, hematocrit content, and incubation times) were varied to obtain the lowest hemolysis. The results for different lipid exchange trials with different conditions and the measured hemolysis are reported in the Supplementary Table S1. The optimized conditions for MαCD-mediated lipid exchange with RBCs and the respective hemolysis values are reported in Table 1. These optimized parameters were used in all remaining experiments. Both MαCD concentrations utilized (20 mM and 40 mM) were capable of mediating the lipid exchange and the smaller one was selected for the remainder of the studies. This was due to the observation that high MαCD concentrations would cause a long dark smeared band on TLC plates. The final analysis of the extracted lipids required three different headgroups of exogenous lipids (as discussed in the later sections), therefore brain SM (bSM), 1-palmitoyl-2-oleoyl-glycero-3-phosphocholine (POPC), and 1-palmitoyl-2-oleoyl-sn-glycero-3-phospho-L-serine (POPS), which showed the lowest hemolysis, were selected.

**Table 1:** The optimized conditions used for the lipid exchange process.

Loaded lipid	MαCD conc. (mM)	Hematocrit conc. (%)	Incubation time (h)	Lipid conc. (mM)	Hemolysis (%)
bSM	20	15	1	0.375	2.2 ± 1.7
				0.75	2.6 ± 1.3
POPC	20	5	1	0.375	0.9 ± 1.1
				0.75	2.1 ± 1.9
POPS	20	5	1	0.75	0
				1.5	0

The hemolysis values are presented as mean ± standard deviation of at least 6 experiments.

### Confocal microscopy images confirm that the delivered lipids are in the outer leaflet

**Figure 1:**
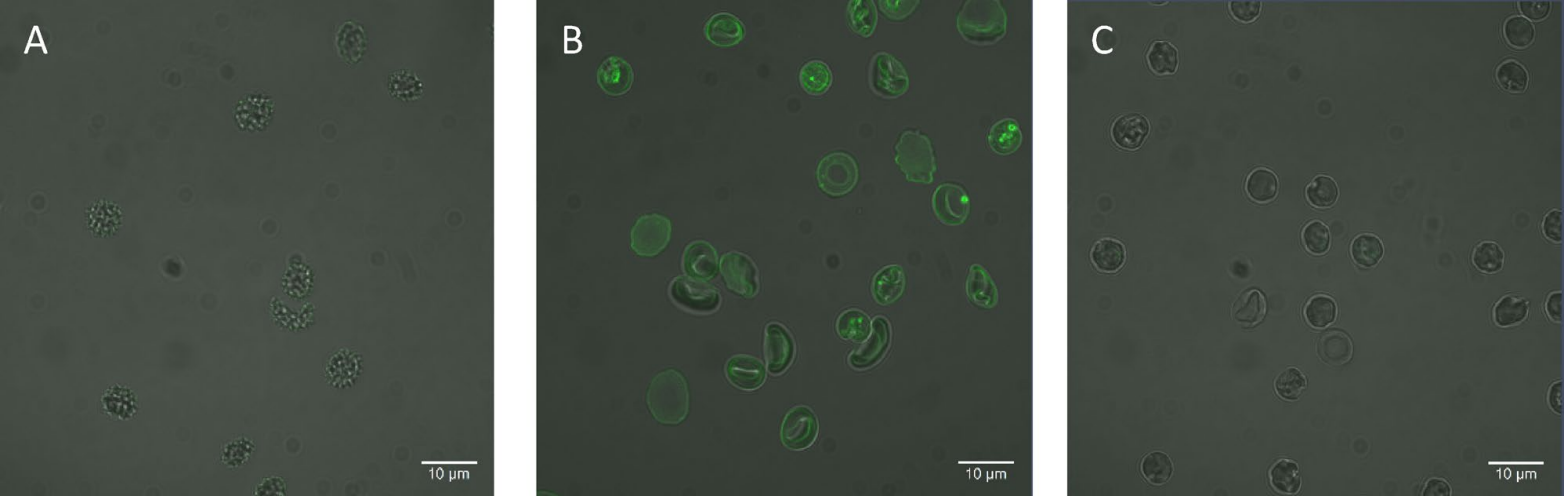
Confocal microscopy images of RBCs. The fluorescence of NBD is shown in green. (**A**) Cells incubated with 1:19 NBD-PE:POPS molar ratio in the absence of MαCD, (**B**) Cells incubated with 1:19 NBD-PE:POPS molar ratio in the presence of MαCD, and (**C**) Cells incubated with 1:19 NBD-PE:POPS molar ratio in the presence of MαCD and treated with 50 mM of sodium dithionite, to quench the NBD fluorescence, immediately before imaging.

To confirm that the exogenous lipids delivered using MαCD were primarily in the cell membrane outer leaflet, confocal microscopy was utilized. A mixture of POPS and the fluorescent lipid, 1,2-dipalmitoyl-sn-glycero-3-phosphoethanolamine-N-(7-nitro-2-1,3-benzoxadiazol-4-yl) (NBD-PE), with a molar ratio of 19:1 POPS:NBD-PE was mixed with 20 mM of MαCD and incubated with RBCs. As a control, RBCs were also incubated with the same lipid mixture without MαCD. Cell incubation with the lipid mixture in the absence of MαCD resulted in negligible fluorescence signal in the cells (Fig. 1A). In contrast, lipids delivered using MαCD resulted in significant fluorescence in the RBC membrane (Fig. 1B), indicating that the lipid exchange only occurred in the presence of MαCD.

In order to evaluate whether the delivered lipids were in the outer leaflet, NBD quenching was used. Sodium dithionite is a relatively membrane impermeable quencher, which cannot penetrate the cell membrane in short incubation times^4, 39^. The NBD molecules conjugated to the headgroup of NBD-PE are therefore only available to sodium dithionite if they are in the outer leaflet, as previously shown by Li et al.^4^. Adding sodium dithionite just prior to imaging, to cells in which NBD-PE was delivered, significantly diminished the NBD fluorescence as shown in Fig. 1C. Almost no intracellular fluorescence could be observed after quenching, indicating that the delivered lipids were primarily delivered to the membrane outer leaflet. It should be noted that sodium dithionite did not abolish membrane asymmetry. This was evidenced by monitoring the level of PS in the membrane outer leaflet of RBCs, as a measure of loss of membrane asymmetry, using lactadherin-FITC staining (Figure S3).

### Lipids were extracted from and delivered to the membrane outer leaflet

Qualitative analysis of the lipids in the supernatant was performed using Thin Layer Chromatography (TLC). To this aim, the supernatant containing the extracted lipids, was subject to lipid extraction using the procedure of Bligh and Dyer^40^ and the resulting lipids were loaded on TLC plates. While the lipid exchange is reported to replace all lipids in the outer leaflet, not all of the lipids that are loaded in MαCD are delivered to the cell during the exchange^4^. This results in the presence of excess amounts of lipids in the supernatant of the lipid exchange mixture at the end of process. Therefore a thick band, corresponding to the loaded lipid, was always present in the TLC plates.

**Figure 2:**
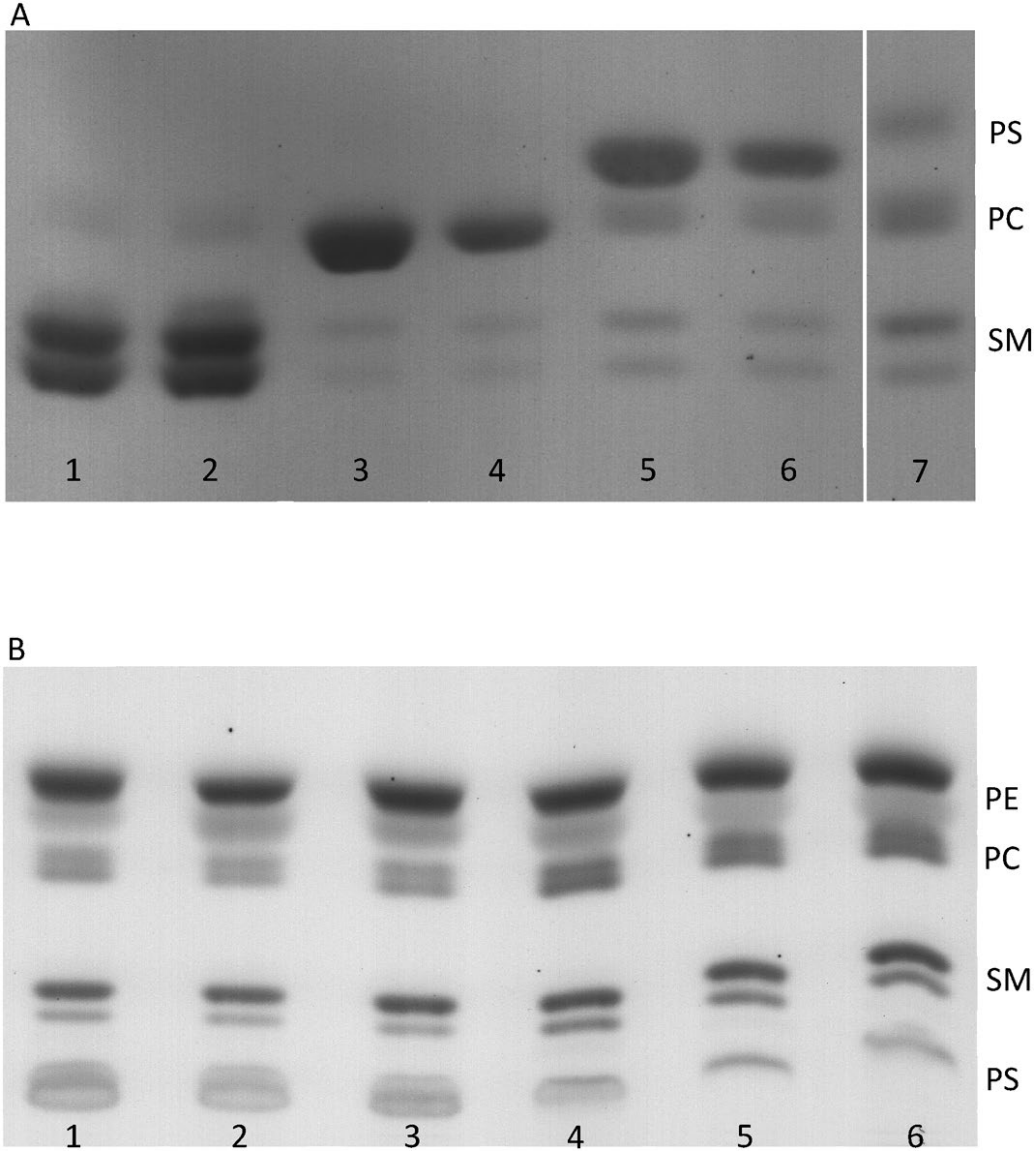
(**A**) Thin layer chromatography of the lipids in the supernatant of the lipid exchange process. The concentration of MαCD was 20 mM. The loaded lipids were present in excess in the supernatant of the exchange process, causing large dark bands on the TLC plate. In lanes 1 and 2, the loaded lipid was bSM at 0.375 mM and 0.75 mM, respectively. In lanes 3 and 4 the loaded lipid was POPC at 0.375 mM and 0.75 mM, respectively. In lanes 5 and 6 the loaded lipid was POPS at 0.75 mM and 1.5 mM, respectively. The concentration of loaded lipids in the odd numbered lanes is twice the concentration of loaded lipids in the even numbered lanes. Lane 7 shows bands for total phospholipid lipid extraction of RBCs. (**B**) Thin layer chromatography of total lipid extracts of RBCs after the exchange. In the first three lanes, RBCs from three different donors were exchanged with POPS (at a concentration of 0.75 mM). Lanes 4–6 are total lipid extracts of RBCs from the same donors as in lanes 1–3, respectively. Note that a different mobile phase was used for each plate, and therefore bands appear in a different order. The uncropped version of both TLC plates are presented in Supplementary Fig. S2.

Figure 2A shows the TLC plate for the experiments performed with the conditions mentioned in Table 1. Analogously to that table, supernatant lipids are presented after extraction with two different concentrations of lipids loaded in MαCD (note that this concentration is different for POPS compared to bSM and POPC). When the exchange was performed with bSM loaded into MαCD (first and second lanes), dark bands for bSM and distinct bands for PC were observed. The observed PC bands in the first two lanes show the extracted PC from the outer leaflet, while the extracted SM is masked under the dark band of the excess bSM in the supernatant. When POPC was the loaded lipid (third and fourth lanes), clear double bands (due to the high abundance of two SM species with different acyl chain lengths^41^) were observed for the extracted SM from the outer leaflet, with the extracted PC being masked under the dark bands formed due to the excess POPC available. The absence of any bands representing PS in the first four lanes indicates that the exchange process extracts lipids only from the membrane outer leaflet and the inner leaflet lipids remained intact. When the exchange was performed with POPS loaded into MαCD (fifth and sixth lanes), double bands for the extracted SM and a single band for the extracted PC were observed. The PS bands in this case only represent the excess POPS in the supernatant and not PS extracted from the cells. This is evidenced by the fact that the ratio of intensity of SM over PC bands in the fifth and sixth lanes is constant, while the concentration of the loaded POPS is halved. The last lane shows total phospholipid composition of untreated RBCs as a control. Bands for SM, PC, and PS can be observed, a thick band is not observed in the control given that no exogenous lipids were present in this case.

Not all lipids were capable of maintaining the integrity of the membrane during the exchange. An example of this was observed when the exchange process was repeated by loading the synthetic lipid SM 35:1 in MαCD. This SM species is not naturally present in cells, which allows for clearly distinguishing the loaded lipids from the extracted lipids. Supplementary Fig. S1 shows the TLC plate of the lipids in the supernatant after the exchange for this set of experiments. The two lanes depicted here represent the lowest hemolysis occurred during the exchange for this lipid. It can be seen that even when the lowest hemolysis is recorded (second lane), PS bands are present. As PS is extensively reported to be in the inner leaflet, its presence in the supernatant can be a result of disruption of the RBCs and extraction of the inner leaflet lipids.

Analysis of total cellular lipids, before and after exchange with POPS, was performed using TLC. Figure 2B shows the TLC plate for total lipid extracts of untreated and lipid-exchanged RBCs from three separate donors. The RBCs collected from the donors were exchanged with POPS and lipids from untreated RBCs from the same donors were included as control. Lanes 1–3 represent the lipid-exchanged RBCs and compared to lanes 4–6, significant changes in the intensity of the bands for PS, SM, and PC can be observed. As the delivered phospholipid was POPS, a thick PS band was observed, while bands for SM and PC became faint compared to the controls. The PE bands did not show any changes. This is because PE is primarily located in the membrane inner leaflet and is thus inaccessible to MαCD. The bands in each lane were quantified and normalized with respect to cholesterol content in that lane (note that MαCD does not extract cholesterol). The efficiency of the exchange process was calculated as: (normalized intensity of lipid band before exchange—normalized intensity of the lipid band after exchange)/(normalized intensity of the lipid band before exchange). The results indicated an exchange efficiency of 50% to 55% for PC and 45% to 50% for SM. Since the majority of SM is expected to be in the outer leaflet^2, 5^, this extraction efficiency is lower than expected. This could be due to the strong interaction of SM with cholesterol, making SM less accessible to the cyclodextrin (further explained in the Discussion).

### Quantitative analysis of phospholipids in the RBCs after exchange

Mass spectrometry was used to provide a quantitative characterization of the RBC membrane phospholipid composition. For these experiments, lipid exchange was performed by loading POPS (PS 34:1) in MαCD and using mass spectrometry to analyze total lipid extraction of RBCs. The logic behind the selection of POPS for loading in MαCD was twofold: (1) POPS exists in RBCs only in trace amounts and thus does not confound the results of the mass spectrometry analysis^24^ and (2) PS species reside only in the inner leaflet of RBCs, therefore the POPS would not mask the lipids that reside in the outer leaflet.

**Figure 3:**
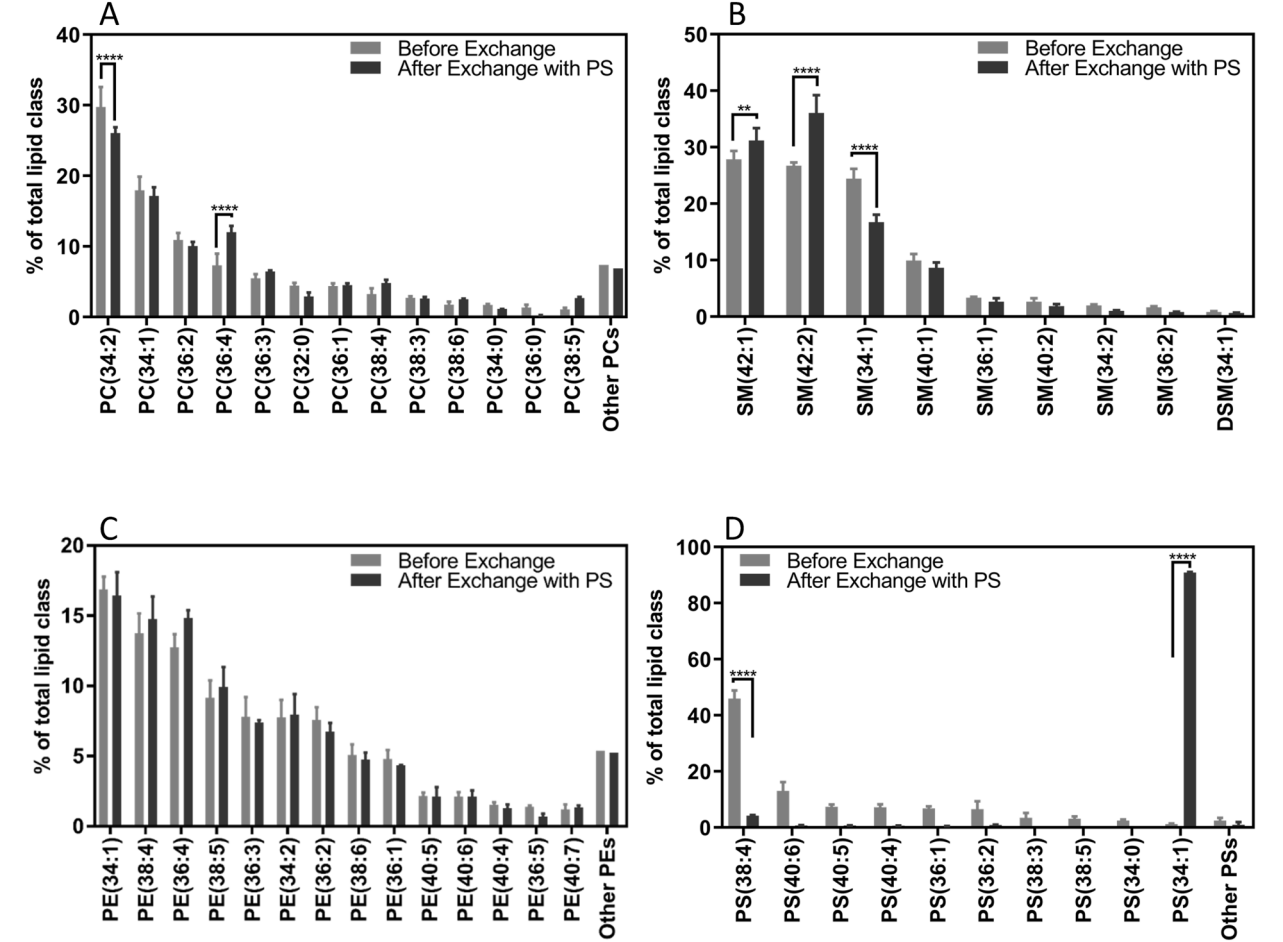
Abundance of phospholipids species in the RBCs before and after exchange with POPS. The breakdown of phospholipid species in terms of molar percentages for each species before and after the lipid exchange is shown individually for (**A**) PC species, (**B**) SM species, (**C**) PE species, and (**D**) PS species. Species with lower than 1% in abundance were summed together and presented as other lipids. Results are presented as mean ± standard deviation of 3 different blood samples. Two way ANOVA analysis, was performed. **p value < 0.01, ****p value < 0.0001.

Figures 3A–D show a breakdown of lipid species of each lipid headgroup for the exchanged RBCs compared to untreated cells. The lipid species are sorted from highest to lowest based on their content in the untreated cells. Of the PC species, PC 34:2 and PC 36:4 and of the SM species, SM 42:1, SM 42:2, and SM 34:1 experience significant changes after the exchange (Fig. 3A,B). This is attributed to the fact that SM and PC are the main constituents of the outer leaflet and are thus extracted during exchange. It can be observed from Fig. 3C that no species of PE has had a significant change after the exchange. This is because PE resides in the inner leaflet and is not accessible to MαCD. Finally, Fig. 3D shows a significant increase in PS 34:1, the species loaded in MαCD, which confirms the delivery of this lipid to the cell. Note that in this case, the reduction of PS 38:4 and other abundant PS species is not due to their extraction, but due to the fact that the total amount of cellular PS has significantly increased, resulting in a significant decrease in the molar percentage of these cells.

### The phospholipid composition of the outer leaflet

Similar to the TLC experiments, the presence of excess exogenous lipids in the supernatant after the exchange poses an obstacle in providing a quantitative breakdown of lipids in the membrane outer leaflet. To resolve this issue, all entries for the exogenous lipids loaded in MαCD (for instance bSM) were removed during data analysis. Then, the lipid content of different species of SM was averaged from the results obtained for samples exchanged with the other two lipids (POPC and POPS). The same analysis was performed for PC and PS as well. After averaging all values for each species, the composition of the phospholipids in the outer leaflet was obtained.

**Figure 4:**
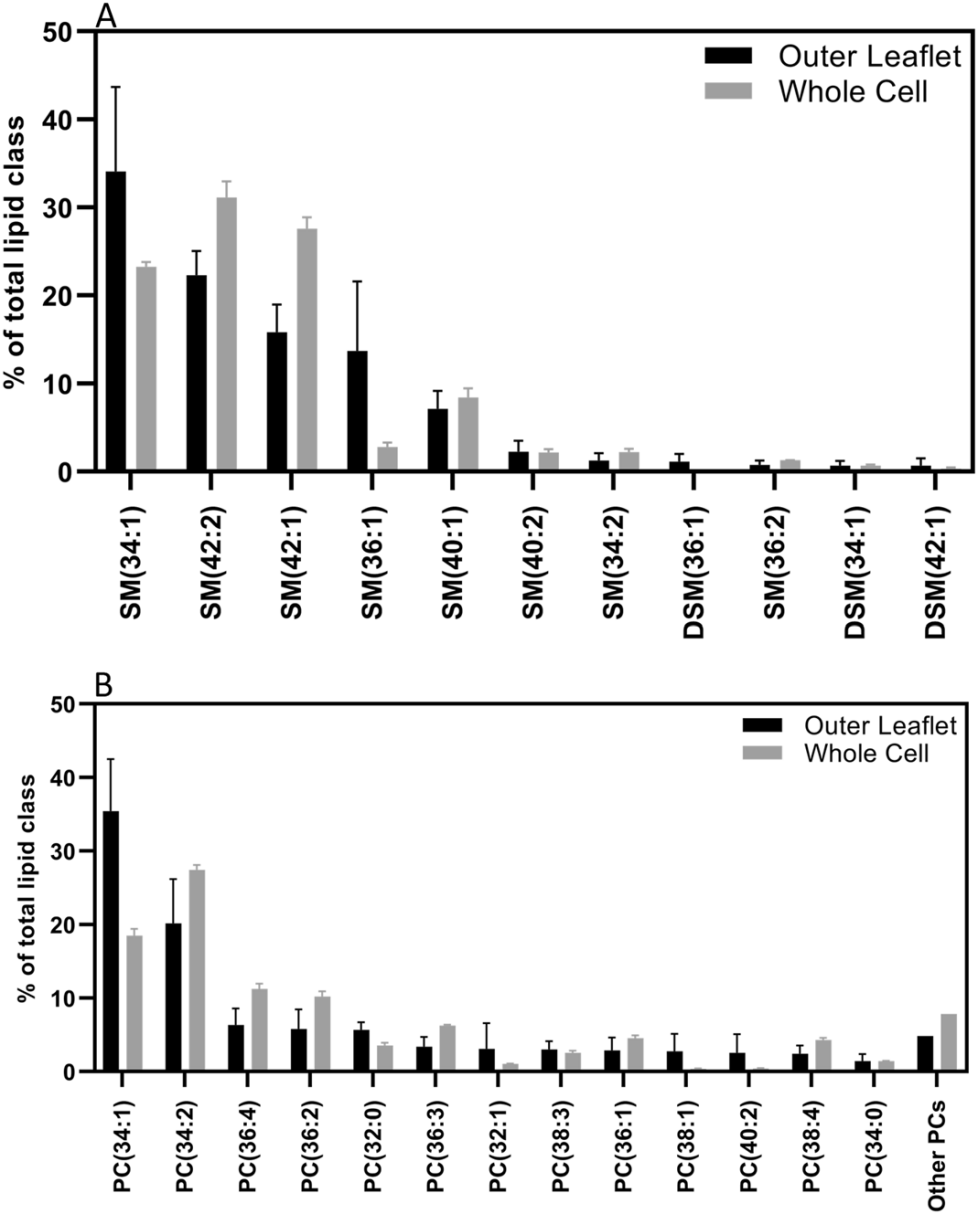
Abundance of (**A**) PC and (**B**) SM species in the outer leaflet of RBCs compared to the abundance of the same species in total lipid extract of RBCs. PC species with lower than 1% in abundance were summed together and presented as other PCs. Results are represented as mean ± standard deviation of three different blood samples.

Figure 4 shows the breakdown of lipid species for two of the most abundant lipids in the outer leaflet of RBCs, PC and SM. It can be seen that PC 34:1 and SM 34:1 are the most abundant PC and SM species, respectively. Among the SM species, the top three species were SM 34:1, SM 42:2, and SM 42:1, in that order (Fig. 4A). This is in complete agreement with the recent results reported by Lorent et al.^5^, based on the enzymatic degradation method. The most abundant PC species were PC 34:1, PC 34:2, and PC 36:4, and PC 36:2, in that order (Fig. 4B). These results show good consistency with the findings of Lorent et al., but minor differences are also observed as that study ranks PCs in the following order in terms of abundance: PC 34:2, PC 34:1, PC 36:2, and PC 36:4^5^. The abundance of different phospholipid species in total RBC lipid extracts are also consistent with the literature^24^.

### Reconstitution of the outer leaflet of senescent RBCs decreases their clearance by macrophages

The use of MαCD to extract lipids from the outer leaflet of RBCs, without disrupting cell membrane integrity, requires that lipids be delivered to the membrane outer leaflet. This could allow for examination of the role of membrane lipids in RBC function. As a potential application, the use of this method to reduce the uptake of senescent RBCs by macrophages was examined. A hallmark of senescent RBCs is translocation of PS to their outer leaflet^18, 42^. This serves as an ‘eat me’ signal to macrophages, which ingest these cells. While this process is beneficial in the body and preserves RBC homeostasis, it poses an issue in preserving RBCs ex vivo^20, 43^. This is because PS exposure occurs much faster during ex vivo storage^44^, which contributes to the rapid clearance of a large portion of transfused cells by the reticuloendothelial system, if cells are transfused after a certain ex vivo ‘age’ (approximately 42 days). It was examined whether exchanging the outer leaflet lipids of the membrane, to remove the PS, can prevent the uptake of RBCs by macrophages.

**Figure 5:**
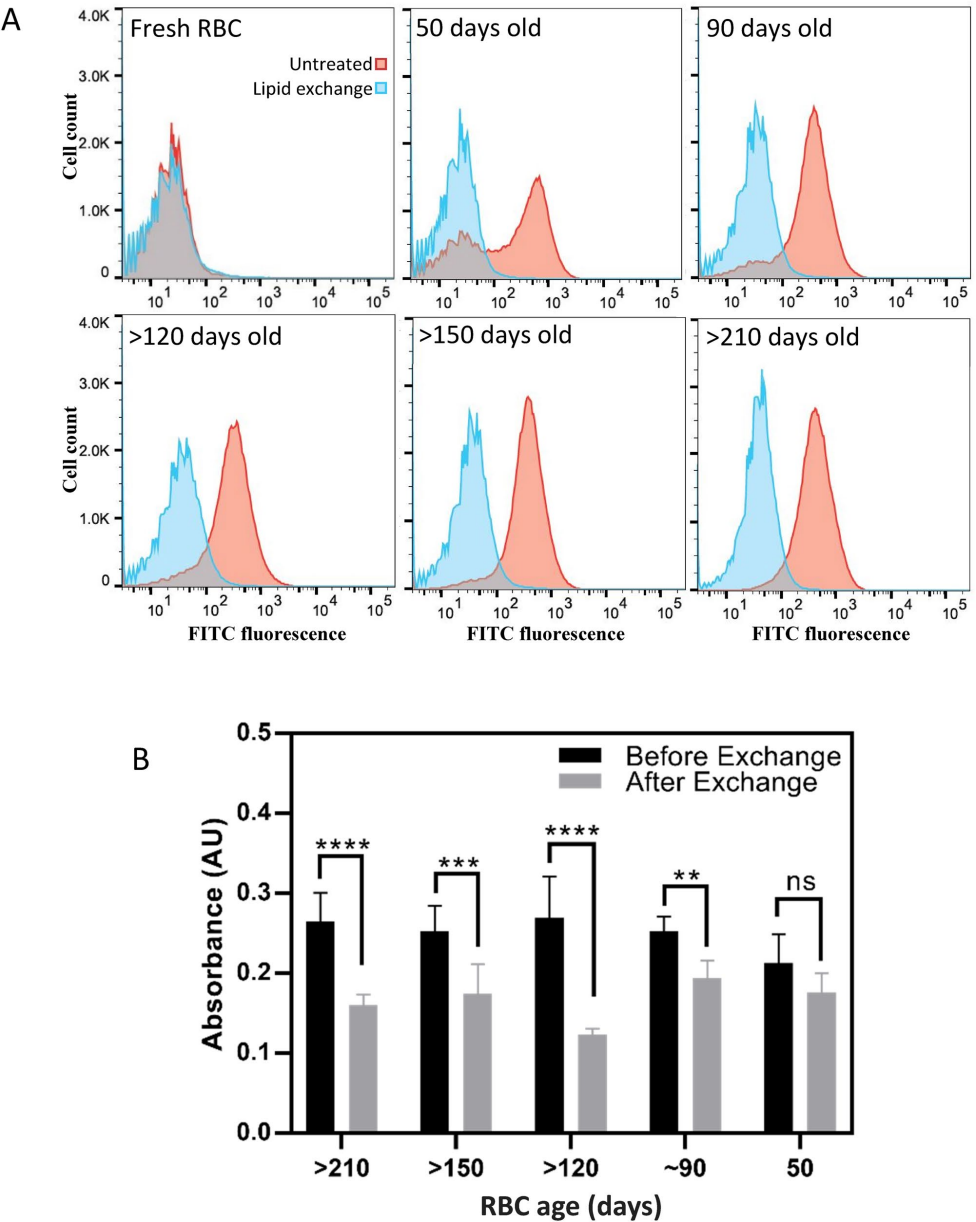
In vitro clearance of senescent RBCs before and after exchange with 1:1 bSM:POPC. (**A**) Flow cytometry results of senescent RBCs before and after lipid exchange. The x-axis depicts the fluorescence intensity of FITC, conjugated to lactadherin, a known PS binding molecule. The red curve represents untreated cells and the blue curve represents the cells with exchanged outer leaflet. (**B**) In vitro macrophage uptake of senescent RBCs before and after exchange. Results are presented as mean ± standard deviation of three independent experiments. Two way ANOVA analysis was performed. **p value < 0.01, ***p value < 0.001, ****p value < 0.0001.

For these experiments, whole blood was acquired with ACD (a mixture of citric acid, sodium citrate, and dextrose) as anti-coagulant and stored in 4 °C to be aged. Aging resulted in the presence of PS in the outer leaflet of RBCs, as measured by flow cytometry of FITC-conjugated lactadherin, a protein known to bind to PS^45^. Significant PS externalization can be observed after 50 days, which is in good agreement with the literature^17, 44, 46^ and was further increased at 90 days after which it plateaued (Fig. 5A, red histograms). All RBCs were then subjected to a membrane lipid exchange, replacing the outer leaflet lipids with 1:1 bSM:POPC. After lipid exchange, the binding of FITC-conjugated lactadherin to the senescent RBCs was reduced to that of fresh RBCs, showing efficient replacement of PS in the outer leaflet (Fig. 5A).

RBCs, before and after the lipid exchange, were then incubated with THP-1 derived macrophages and their uptake by macrophages was measured using a calorimetric method^47^. This method is based on the detection of hemoglobin inside the macrophages, with higher absorbance values indicating higher hemoglobin content. Lipid exchange resulted in substantial reduction of RBC uptake by macrophages, which was statistically significant in all RBCs aged for 90 days and longer (Fig. 5B). The difference in uptake for cells aged for 50 days, which expressed the lowest amount of PS in the outer leaflet, was not statistically significant. Although even in those cells, lipid exchange resulted in a reduction in macrophage uptake.

## Discussion

While the phospholipid asymmetry of the RBC plasma membrane has been known for more than 40 years, methods to analyze the outer leaflet lipid composition still depend on enzymatic degradation of the outer leaflet lipids. In this study, we have developed a method, based on cyclodextrin-mediated lipid exchange to characterize the outer leaflet phospholipids of RBCs. This method, which builds up on previous studies to determine outer membrane leaflet composition in cancer cells^4^, does not perturb membrane integrity and provides results that are compatible with the enzymatic degradation method. Importantly, the delivery of lipids to the outer leaflet of RBCs facilitated by this method can be useful in ex vivo preservation of RBCs, by reconstituting their membrane and preventing their uptake by macrophages.

The method in the current study is based on cyclodextrin-mediated lipid exchange to extract the outer leaflet of RBCs. The pioneering study of Li et al. had shown that this method can efficiently replace the outer leaflet of a variety of mammalian cells (A549, MDA-MB-231, COS 7, and BxPC-3 cells) with an exogenous phospholipid^4^. Here, this process was optimized to cause the lowest level of damage to the membrane integrity of RBCs during lipid extraction. Similar to the methods of Li et al., exogenous lipids were loaded in cyclodextrin to prevent RBC membrane damage. Interestingly, both inner (POPS) and outer leaflet lipid headgroups (bSM and POPC) were able to maintain membrane integrity when delivered to the outer leaflet (Table 1). The acyl chain length and unsaturation state of the lipids are the two most important parameters in MαCD complex formation with the lipids^48, 49^. Thus, complex formation of MαCD with POPC and POPS is expected to be similar, due to the similarity of their chain structure. Given the fatty acid distribution of bSM, this lipid is also expected to complex with MαCD to a similar extent. Thus, while using POPS as the exogenous lipid generally led to lower hemolysis values (Table S1), this does not necessarily mean that this lipid complexes more efficiently with MαCD compared to SM and PC. A better understanding of the mechanisms of complex formation and lipid exchange is needed to tease out why certain lipids and lipid concentrations are more efficient at maintaining RBC membrane integrity.

Quantification of the band intensity on TLC plates showed that the exchange process can remove 50–55% of PC and 45–50% of the SM, with exogenous lipids being delivered to the membrane outer leaflet (Fig. 2). Lorent et al. reported that about 60% of the PC and 90% of the SM reside in the outer leaflet^5^. Thus, the moderate exchange efficiency for SM, especially compared to PC (which shows near complete removal) is somewhat surprising. One potential explanation for this lower extraction efficiency is the favorable interaction of SM with cholesterol^50, 51^. The affinity order of cholesterol for phospholipids is in the order of SM > PC > PE^52^. In fact, degradation of membrane SM, but not PC, using the enzyme sphingomyelinase, has been shown to result in increased extraction of cholesterol from the membrane of fibroblasts using MβCD^53^. Thus, it is possible that the high concentration of cholesterol in the outer leaflet of RBCs hinders cyclodextrin-mediated extraction of SM as well. Experiments with cholesterol oxidase, to reduce the amount of membrane cholesterol, followed by membrane lipid extraction using cyclodextrin, or experiments with asymmetric vesicles, with varying degrees of cholesterol and SM in the outer leaflet could help shed light on this issue.

Here, a note on cholesterol asymmetry in the RBC membrane is also warranted. Cholesterol constitutes approximately 40%^5^ of the plasma membrane lipids of RBC and is known to distribute asymmetrically in the plasma membrane. While there are reports of cholesterol preferentially residing in the outer leaflet due to its affinity to saturated chains of SM and PC, there are also studies suggesting that PE and PS can draw cholesterol to the inner leaflet^54^. The rapid flip-flop rate of cholesterol^55, 56^, along with the differences in its distribution in different cells have led to conflicting reports on its distribution^54, 57^. Regardless, evidence on cholesterol affinity for certain phospholipids^52^ and toward more ordered membranes^58^ exist. Thus, it is possible that the cholesterol in the outer leaflet interacts more with the SM, due to its long and saturated acyl chains, which further inhibits SM extraction from the membrane.

To obtain the lipid composition of the outer leaflet, the membrane of RBCs were exchanged with bSM, POPC, and POPS separately and the extracted lipids were analyzed using mass spectrometry. The abundance values for the species of loaded lipids were replaced by averaging the abundance values of the species of that lipid from samples exchanged using the other two lipids. This allowed for removal of the values for the excess loaded lipid that would confound the mass spectrometry analysis otherwise. Here, the drawbacks of using cyclodextrins to probe plasma membrane lipids should also be pointed out. Extensive extraction of lipids from the plasma membrane by cyclodextrins can lead to cell lysis^59^. While this can be prevented by loading cyclodextrins with exogenous lipids^4, 38^, this process will likely need to be optimized individually for each cell, increasing the burden for such analyses. In addition, the possibility that cyclodextrin might be biased toward certain lipid structures cannot be ruled out. For example, while strong bias toward certain lipid headgroups, except for phosphatidylinositol, are not generally observed^35^, the chain length and saturation, as noted above, are known to affect cyclodextrin-lipid complex formation^36, 48, 49^.

The composition of the individual leaflets of the plasma membrane of RBCs was first characterized over 40 years ago by Verkleij et al.^2^. That study showed that SM and PC constitute more than 85% of phospholipids in the outer leaflet, with the rest being PE. In contrast, the inner leaflet has a total of less than 25% SM and PC, more than 45% of PE, and more than 25% PS. It is worth mentioning that the reported bar graphs of Verkleij et al.^2^ do not add up to 100%. The methods used in that study were not capable of reporting the composition of other lipids, now known to reside in the membrane (e.g. lyso lipids). Those methods were also not capable of providing a detailed breakdown of phospholipid acyl chain structure in each leaflet. Recently, Lorent et al. performed similar experiments, but analyzed the results using mass spectrometry and were able to present the breakdown of each phospholipid in the membrane as well^5^. Contradictory to the results by Verkleij et al.^2^, Lorent and colleagues reported negligible PE in the outer leaflet^5^. Importantly, while the methods are clearly distinct, there are remarkable similarities in the breakdown of outer leaflet PC and SM between the current study and the study of Lorent et al. Here, the most abundant PC and SM species in the outer leaflet are PC 34:1, PC 34:2, SM 34:1, and SM 42:2 (Fig. 4), which is consistent with the results provided by Lorent et al. Interestingly, the abundance of SM species in the outer leaflet is also completely consistent between the two studies. Despite striking similarities between results from the two methods, differences are also observed. For example, our results show PC 34:1 as the most and PC 34:2 as the second most abundant PC species in the outer leaflet, while the reverse is reported by Lorent et al. These minor differences could be due to the fact that cyclodextrin favors more saturated lipids over polyunsaturated ones^35^. However, as the abundance of the SM species shows, the lipid exchange was capable of extracting the SM species from the outer leaflet, in an unbiased manner.

While the characterization of the membrane outer leaflet lipid composition can be important in the context of membrane biology and RBC function in health and disease, an important application of this method could be to deliver lipids of choice to the outer leaflet of the RBC membrane. Here, this method was used to reconstitute the membrane of senescent RBCs to alter their interactions with macrophages. As RBCs age, PS translocates to the membrane outer leaflet, this is a signal for initiation of erythrophagocytosis (i.e. uptake of RBCs by phagocytes), which, among other mechanisms, can occur through the binding of bridging molecules to PS, resulting in uptake through integrins or TAM receptors^19, 20^. When exchanging the lipids with 1:1 bSM:POPC, the PS level, regardless of the age of the RBCs, can be lowered to that of the fresh RBCs, resulting in a significant reduction of RBC uptake by macrophages. Since MαCD does not interact with RBC proteins^59^, this significant reduction in uptake is expected to be primarily due to the removal of PS. While in the current study PS removal has been used to show one application of cyclodextrin-mediated lipid exchange in RBCs, we anticipate such lipid manipulations to be more widely used in studies of RBC membrane biology and to find applications in altering the RBC membrane to affect cell–cell interactions.

## Methods

### RBC maintenance and cell culture

De-identified human whole blood was obtained in acid citrate dextrose (ACD) as anti-coagulant, from ZenBio (Research Triangle, NC) and was stored at 4 °C. THP-1 monocytes (ATCC TIB-202) were cultured in RPMI-1640, supplemented with 10% FBS, and were cultured in an incubator at 37 °C with 5% CO_2_.

### Phospholipid exchange using cyclodextrins

Different amounts of various phospholipids (as reported in Table 1 and Supplementary Table S1) were first dried out of their organic solvents using a SpeedVac (Thermo Fisher Scientific, Waltham, MA). The dried lipids were then heated to a temperature above their melting point (70 °C) and rehydrated for 5 min with pre-warmed Dulbecco’s Phosphate Buffered Saline (DPBS) at the same temperature. The mixture was then vortex-mixed at 3,000 rpm for 10 min to form lipid vesicles. Separately, methyl-α-cyclodextrin (MαCD) was dissolved in DPBS at a final concentration of 80 mM. The resulting turbid solution was centrifuged at 14,000 g for 5 min to obtain a clear solution without any precipitates. Desired volumes of the MαCD solution were added to the lipid vesicle suspension to obtain the final concentrations of MαCD and lipids as needed for the experiments. The MαCD/lipid solution was briefly mixed by pipetting and incubated at room temperature for 30 min. Packed triple washed RBCs were transferred to the experiment vials and desired amount of MαCD/lipid solution was added to obtain the final hematocrit concentrations mentioned in the tables. All hematocrit contents are on a volume basis. The mixture was incubated at room temperature without shaking for different times as mentioned in Supplementary Table S1. After incubation, the suspension was laid on 2 mL of 10% (w/v) sucrose solution in DPBS and was centrifuged at 750 g for 5 min. The difference in the density resulted in precipitation of the RBCs at the bottom of the tube, while MαCD, excess exogenous lipids, as well as the extracted lipids were floated to the top of the sucrose solution. This protocol was exactly followed anytime the exchange was performed.

### Measuring the hemolysis of RBCs

Presence of hemoglobin in the exchange solution was used as a measure of RBC membrane disruption (hemolysis). To monitor the amount of hemolysis caused by the exchange, at the end of the incubation period, the suspension was laid on 2 mL of 10% (w/v) sucrose solution in DPBS and was centrifuged at 750 g for 5 min, the lipids and MαCD were removed from the top, 200 μL of the supernatant was collected, and its absorbance at 541 nm was measured using a plate reader. RBCs suspended in DPBS and water for the same incubation period, at the same hematocrit concentrations as the ones used for exchange experiments, were used as negative and positive controls for hemolysis, respectively. Both controls were also laid on 2 mL of 10% (w/v) sucrose solution in DPBS and were centrifuged at 750 g for 5 min prior to measuring their absorbance.

### Identification of extracted and delivered phospholipids

As phospholipid exchange proceeds, exogenous phospholipids are delivered to the membrane, replacing the endogenous phospholipids of the outer leaflet of RBCs. To identify the phospholipids in the supernatant of the exchange process, the top 1 mL of the supernatant was collected after centrifugation and the lipids were extracted using the Bligh & Dyer method^40^. According to the specific volumes provided by this method, solutions of 2:1 methanol:chloroform, chloroform and water were added to the samples, with vigorous vortex-mixing after each addition. The final milky suspension was centrifuged at 2000 g for 5 min and the bottom phase was collected using a Pasteur pipette, with positive pressure (bubbling through the top phase) to avoid contamination. The collected solution was then dried using a SpeedVac and the dried lipids were then re-suspended in 100 μL of chloroform. The latter was then carefully loaded to a high performance Thin Layer Chromatography (TLC) plate. The plate was then placed in a TLC tank, containing a mobile phase of 4:8:38:50 acetic acid:water:methanol:chloroform. When the solvent front reached the top of the plate, the plate was taken out of the tank and air dried. The plate was then sprayed with a solution of 3% cupric acetate in 8% phosphoric acid and air dried again. Finally, the plate was charred in an oven at 185 °C for 5–10 min^30^. To identify the phospholipids delivered to the RBCs, the supernatant of the exchange was discarded after centrifugation and the RBCs were lysed with water. Untreated RBCs from the same donors as the ones for the exchange was collected to the same hematocrit content and were lysed in water. The lipid extraction and TLC experiments were performed exactly as described earlier, with the exception of the mobile phase which was 4:25:65 ammonium hydroxide:methanol:chloroform for this plate. The solvent was changed to obtain a better separation between all the phospholipids as well as cholesterol. The plate was also air dried, sprayed, and charred as described earlier. The bands in each lane of this plate were quantified and normalized with respect to cholesterol.

### Confocal microscopy of RBCs after exchange with a fluorescent lipid

Delivery of phospholipids to the membrane was studied using fluorescence. A quenching agent was used to diminish the fluorescence and evaluate whether the lipids are delivered to the membrane outer leaflet. Organic solutions of a fluorescent phospholipid (NBD-PE) and POPS were mixed to a molar ratio of 1:19 NBD-PE:POPS (final concentration of lipids was 0.75 mM). The other steps prior to the exchange experiment were performed as mentioned before. After the exchange, the suspension was laid on 2 mL of 10% (w/v) sucrose solution in DPBS and was centrifuged at 750 g for 5 min, the precipitated RBCs were collected and re-suspended in 100 μL of 10% (w/v) sucrose in DPBS solution. Seven μL of the suspension was loaded onto a glass slide and imaged using a confocal microscope. Sodium dithionite solution was made freshly and added to an aliquot of the exchanged RBCs to achieve a final concentration of 50 mM just prior to loading RBCs onto glass slides and imaging. To assess the effects of sodium dithionite treatment on the RBCs, triple washed expired and fresh RBCs were incubated with 50 mM of sodium dithionite for 2 min, were then centrifuged at 750 g for 5 min, and washed with DPBS. Treated and untreated RBCs were brought to 1% (v/v) hematocrit content, lactadherin-FITC was added to a final concentration of 20 nM to both samples, and the samples were incubated for 30 min at room temperature. The RBCs were then washed twice with centrifugation and the fluorescence of the samples was recorded.

### Mass spectrometry analysis of the lipids

Quantification of the phospholipids and their respective species were performed using mass spectrometry. After exchanging the outer leaflet of RBCs with POPS, the cells were collected, lysed using water to remove the hemoglobin, and a total lipid extraction was performed on the cells using the methods of Bligh and Dyer^40^. The lipids in the supernatant of the exchange process, after separate exchanges with bSM, POPC, and POPS were also extracted using the Bligh & Dyer method. The lipids were then dried, weighed, and used for mass spectrometry. Lipidomics analysis were performed using Mass Spectrometry (MS) at the Kansas Lipidomics Research Center. Direct-infusion electrospray ionization triple quadrupole mass spectrometry was carried out on an API4000 QTrap (ABSciex). Samples contained 0.06 mg of lipid. Phospholipid internal standards were mixed with the samples, and a solvent mixture (chloroform:methanol:300 mM ammonium acetate in water, 30:66.5:3.5) was added to samples to achieve a final volume of 1.2 ml. Samples were analyzed by a series of precursor and neutral loss scans, as described by Narayanan et al.^60^. Minor differences from the Narayanan protocol were that diphytanoyl PE [PE(20:0/20:0)] was substituted for internal standard PE(23:0/23:0), and the amount of internal standard PI(16:0/18:0) was 0.28 nmol. Data were processed as described by Narayanan et al.^60^, detecting SM with Pre 184 in positive mode and using the PC standards for PC and SM, except that additional response factors determined by comparing the employed internal standards to SPLASH internal standards (Avanti Polar Lipids) were employed. Pooled quality control samples were included in the analysis to determine analytical precision, but they were not used to correct the sample data. Lipidomics data were used as obtained. The only changes made in the data were removing the entries that were below the detection limit of the instrument. After that, the species of each phospholipid headgroup were summed together to obtain the total abundance of each headgroup. Two-way ANOVA was performed on the results to obtain the significance of changes compared to the untreated RBCs as control.

### Detection of PS on the outer leaflet before and after exchange

Presence of PS in the outer leaflet of the mammalian cells is attributed to the apoptosis of the cells. Milk fat globule epidermal growth factor 8 protein (a.k.a. lactadherin) conjugated with FITC was used to detect the PS in the outer leaflet of red bool cells^45^. Senescent RBCs (aged more than 60 days) were washed three times, brought to 1% hematocrit content, and were then incubated with lactadherin-FITC to a final concentration of 20 nM for 30 min. The RBCs obtained from the same donors were exchanged with 1:1 bSM:POPC as described before, the cells were washed and collected, and were incubated with the same concentration of lactadherin-FITC. A flow cytometer was used to detect the FITC intensity for 100,000 events.

### Macrophage uptake of senescent RBCs

Macrophages were derived from THP-1 monocytes as described by Starr et al.^61^. Briefly, the THP-1 cells were incubated for 48 h prior to use with 20 ng/mL of Phorbol 12-Myristate 13-Acetate (PMA), in a 96 well plate to differentiate to macrophages. The macrophages were washed twice with warm media, then incubated with exchanged and untreated senescent RBCs (separately) at a ratio of 1:20 macrophages:RBCs for two hours. The uptake of RBCs is measured using a calorimetric method based on specific oxidation of 2,7-diaminofluorene (DAF) by hemoglobin^47^. The macrophages were washed two times with media to remove the excess RBCs and then the surface bound RBCs are lysed using a hypotonic solution (0.25% NaCl) for 3 min. The wells were washed twice again using media to remove any RBCs or free hemoglobin in the well. The remaining cells were then lysed using a solution of 0.2 M Tris–HCl in 6 M urea. A solution of 100 mg DAF in 10 mL of 90% glacial acetic acid in water was then made (DAF stock). 1 mL of DAF stock solution was added to 10 mL of 0.2 M Tris–HCl in 6 M urea solution and 100 μL of 30% hydrogen peroxide was added. 100 μL of the final product was added to each well, and after 5 min of incubation, the absorbance of each well was measured at 620 nm.

## Supplementary information

Supplementary Information.

## References

[1] Op den Kamp JA (1979). Lipid asymmetry in membranes. Annu. Rev. Biochem..

[2] Verkleij A (1973). The asymmetric distribution of phospholipids in the human red cell membrane. A combined study using phospholipases and freeze-etch electron microscopy. Biochim. Biophys. Acta Biomembr..

[3] Vance DE, Vance JE (1996). Biochemistry of Lipids, Lipoproteins and Membranes.

[4] Li G (2016). Efficient replacement of plasma membrane outer leaflet phospholipids and sphingolipids in cells with exogenous lipids. Proc. Natl. Acad. Sci..

[5] Lorent J (2020). Plasma membranes are asymmetric in lipid unsaturation, packing and protein shape. Nat. Chem. Biol..

[6] van Engeland M, Ramaekers FC, Schutte B, Reutelingsperger CP (1996). A novel assay to measure loss of plasma membrane asymmetry during apoptosis of adherent cells in culture. Cytometry.

[7] Fadeel B, Xue D (2009). The ins and outs of phospholipid asymmetry in the plasma membrane: roles in health and disease. Crit. Rev. Biochem. Mol. Biol..

[8] Bevers EM, Comfurius P, Zwaal RF (1983). Changes in membrane phospholipid distribution during platelet activation. Biochim. Biophys. Acta Biomembr..

[9] Vermes I, Haanen C, Reutelingsperger C (2000). Flow cytometry of apoptotic cell death. J. Immunol. Methods.

[10] Moras M, Lefevre SD, Ostuni MA (2017). From erythroblasts to mature red blood cells: organelle clearance in mammals. Front. Physiol..

[11] Kumar A, Gupta C (1983). Red cell membrane abnormalities in chronic myeloid leukaemia. Nature.

[12] Zwaal RF (1989). Loss of membrane phospholipid asymmetry during activation of blood platelets and sickled red cells; mechanisms and physiological significance. Mol. Cell. Biochem..

[13] Yasin Z (2003). Phosphatidylserine externalization in sickle red blood cells: associations with cell age, density, and hemoglobin F. Blood.

[14] Yashar VBB, Barenholz Y, Hy-Am E, Rachmilewitz EA, Eldor A (1993). Phosphatidylserine in the outer leaflet of red blood cells from β-thalassemia patients may explain the chronic hypercoagulable state and thrombotic episodes. Am. J. Hematol..

[15] Wahid, S. T., Marshall, S. M. & Thomas, T. H. Increased platelet and erythrocyte external cell membrane phosphatidylserine in type 1 diabetes and microalbuminuria. *Diabetes Care* **24** (2001). 10.2337/diacare.24.11.2001-a 11679474

[16] Zwaal R, Comfurius P, Bevers E (2005). Surface exposure of phosphatidylserine in pathological cells. Cell. Mol. Life Sci..

[17] Connor J, Pak CC, Schroit AJ (1994). Exposure of phosphatidylserine in the outer leaflet of human red blood cells. Relationship to cell density, cell age, and clearance by mononuclear cells. J. Biol. Chem..

[18] Lang K (2005). Mechanisms of suicidal erythrocyte death. Cell. Physiol. Biochem..

[19] de Back DZ, Kostova EB, van Kraaij M, van den Berg TK, Van Bruggen R (2014). Of macrophages and red blood cells; a complex love story. Front. Physiol..

[20] Klei TR, Meinderts SM, van den Berg TK, van Bruggen R (2017). From the cradle to the grave: the role of macrophages in erythropoiesis and erythrophagocytosis. Front. Immunol..

[21] Kamp D, Sieberg T, Haest CW (2001). Inhibition and stimulation of phospholipid scrambling activity. Consequences for lipid asymmetry, echinocytosis, and microvesiculation of erythrocytes. Biochemistry.

[22] Tinmouth A, Chin-Yee I (2001). The clinical consequences of the red cell storage lesion. Transfus. Med. Rev..

[23] Schroit AJ, Madsen JW, Tanaka Y (1985). In vivo recognition and clearance of red blood cells containing phosphatidylserine in their plasma membranes. J. Biol. Chem..

[24] Leidl K, Liebisch G, Richter D, Schmitz G (2008). Mass spectrometric analysis of lipid species of human circulating blood cells. Biochim. Biophys. Acta Mol. Cell. Biol. Lipids.

[25] Etemadi A-H (1980). Membrane asymmetry A survey and critical appraisal of the methodology II. Methods for assessing the unequal distribution of lipids. Biochim. Biophys. Acta Biomembr..

[26] Krebs JJ (1982). The topology of phospholipids in artificial and biological membranes. J. Bioenerg. Biomembr..

[27] Zwaal R, Roelofsen B, Comfurius P, Van Deenen L (1975). Organization of phospholipids in human red cell membranes as detected by the action of various purified phospholipases. Biochimica et Biophysica Acta (BBA)-Biomembranes.

[28] Demel R, Geurts van Kessel W, Zwaal R, Roelofsen B, Van Deenen L (1975). Relation between various phospholipase actions on human red cell membranes and the interfacial phospholipid pressure in monolayers. Biochimica et Biophysica Acta (BBA)-Biomembranes.

[29] Szejtli J (1998). Introduction and general overview of cyclodextrin chemistry. Chem. Rev..

[30] Cheng H-T, London E (2009). Preparation and properties of asymmetric vesicles that mimic cell membranes effect upon lipid raft formation and transmembrane helix orientation. J. Biol. Chem..

[31] Huang Z, London E (2013). Effect of cyclodextrin and membrane lipid structure upon cyclodextrin–lipid interaction. Langmuir.

[32] London, E. in *Characterization of Biological Membranes: Structure and Dynamics* Ch. 14, 441–464 (2019).

[33] Zidovetzki R, Levitan I (2007). Use of cyclodextrins to manipulate plasma membrane cholesterol content: evidence, misconceptions and control strategies. Biochim. Biophys. Acta Biomembr..

[34] Doktorova M (2018). Preparation of asymmetric phospholipid vesicles for use as cell membrane models. Nat. Protoc..

[35] Vahedi, A. & Farnoud, A. in *Analysis of Membrane Lipids* Ch. 9, (2020).

[36] Tanhuanpää K, Cheng KH, Anttonen K, Virtanen JA, Somerharju P (2001). Characteristics of pyrene phospholipid/γ-cyclodextrin complex. Biophys. J..

[37] Kainu V, Hermansson M, Somerharju P (2010). Introduction of phospholipids to cultured cells with cyclodextrin. J. Lipid Res..

[38] Li G (2019). Replacing plasma membrane outer leaflet lipids with exogenous lipid without damaging membrane integrity. PloS One.

[39] Moreno MJ, Estronca LM, Vaz WL (2006). Translocation of phospholipids and dithionite permeability in liquid-ordered and liquid-disordered membranes. Biophys. J..

[40] Bligh EG, Dyer WJ (1959). A rapid method of total lipid extraction and purification. Can. J. Biochem. Physiol..

[41] Ramstedt B, Leppimäki P, Axberg M, Slotte JP (1999). Analysis of natural and synthetic sphingomyelins using high-performance thin-layer chromatography. Eur. J. Biochem..

[42] Schlegel R, Williamson P (2001). Phosphatidylserine, a death knell. Cell Death Differ..

[43] Hess J (2006). An update on solutions for red cell storage. Vox Sang..

[44] Lu C, Shi J, Yu H, Hou J, Zhou J (2011). Procoagulant activity of long-term stored red blood cells due to phosphatidylserine exposure. Transfus. Med..

[45] Dasgupta SK, Guchhait P, Thiagarajan P (2006). Lactadherin binding and phosphatidylserine expression on cell surface-comparison with annexin A5. Transl. Res..

[46] Dinkla S (2014). Phosphatidylserine exposure on stored red blood cells as a parameter for donor-dependent variation in product quality. Blood Transfus..

[47] Gebran S, Romano E, Pons H, Cariani L, Soyano A (1992). A modified colorimetric method for the measurement of phagocytosis and antibody-dependent cell cytotoxicity using 2, 7-diaminofluorene. J. Immunol. Methods.

[48] Fauvelle F, Debouzy J, Crouzy S, Göschl M, Chapron Y (1997). Mechanism of α-cyclodextrin-lnduced hemolysis. 1. The two-step extraction of phosphatidylinositol from the membrane. J. Pharm. Sci..

[49] Debouzy J (1998). Mechanism of α-cyclodextrin induced hemolysis. 2. A study of the factors controlling the association with serine-, ethanolamine-, and choline-phospholipids. J. Pharm. Sci..

[50] Aittoniemi J, Niemelä PS, Hyvönen MT, Karttunen M, Vattulainen I (2007). Insight into the putative specific interactions between cholesterol, sphingomyelin, and palmitoyl-oleoyl phosphatidylcholine. Biophys. J ..

[51] Tsamaloukas A, Szadkowska H, Heerklotz H (2006). Thermodynamic comparison of the interactions of cholesterol with unsaturated phospholipid and sphingomyelins. Biophys. J ..

[52] Demel R, Jansen J, Van Dijck P, Van Deenen L (1977). The preferential interaction of cholesterol with different classes of phospholipids. Biochem. Biophys. Acta..

[53] Ohvo H, Olsio C, Slotte JP (1997). Effects of sphingomyelin and phosphatidylcholine degradation on cyclodextrin-mediated cholesterol efflux in cultured fibroblasts. Biochimica Et Biophysica Acta (BBA)-Lipids Lipid Metab..

[54] Steck TL, Lange Y (2018). Transverse distribution of plasma membrane bilayer cholesterol: picking sides. Traffic.

[55] Steck TL, Ye J, Lange Y (2002). Probing red cell membrane cholesterol movement with cyclodextrin. Biophys. J ..

[56] Bennett WD, MacCallum JL, Hinner MJ, Marrink SJ, Tieleman DP (2009). Molecular view of cholesterol flip-flop and chemical potential in different membrane environments. J. Am. Chem. Soc..

[57] Devaux PF, Morris R (2004). Transmembrane asymmetry and lateral domains in biological membranes. Traffic.

[58] Ingólfsson HI (2014). Lipid organization of the plasma membrane. J. Am. Chem. Soc..

[59] Ohtani Y, Irie T, Uekama K, Fukunaga K, Pitha J (1989). Differential effects of α-, β-and γ-cyclodextrins on human erythrocytes. Eur. J. Biochem..

[60] Narayanan S, Tamura PJ, Roth MR, Prasad PV, Welti R (2016). Wheat leaf lipids during heat stress: I. High day and night temperatures result in major lipid alterations. Plant Cell Environ..

[61] Starr T, Bauler TJ, Malik-Kale P, Steele-Mortimer O (2018). The phorbol 12-myristate-13-acetate differentiation protocol is critical to the interaction of THP-1 macrophages with Salmonella Typhimurium. PloS One.

