# Supplementary material for: Quantitative analysis of red blood cell membrane phospholipids and modulation of cell-macrophage interactions using cyclodextrins
Source: Sci Rep. 2020 Sep 15;10:15111. doi: 10.1038/s41598-020-72176-3 (PMC7492248; doi:10.1038/s41598-020-72176-3)
Supplement: Supplementary file 1 — Supplementary Information. [file 41598_2020_72176_MOESM1_ESM.docx]

**Quantitative analysis of red blood cell membrane phospholipids and modulation of cell-macrophage interactions using cyclodextrins**

Amid Vahedi^1^, Parnian Bigdelou^2^, Amir M. Farnoud^1,2*^

^1^ Department of Chemical and Biomolecular Engineering and ^2^Biomedical Engineering Program Ohio University, Athens, Ohio 45701, United States

* To whom correspondence should be addressed: Dr. Amir M. Farnoud, Department of Chemical and Biomolecular Engineering, 161 Stocker Center, Ohio University, Athens, OH 45701, Tel.: (740) 593-1426, Fax: (740) 593-0873; E-mail: farnoud@ohio.edu

Table S1. Hemolysis values recorded for different conditions of the exchange process. The values are presented as mean ± standard deviation of at least three independent experiments.

| Loaded Lipid | M𝛂CD Conc. (mM) | Hematocrit Content | Incubation Time | Lipid Conc. (mM) | Hemolysis (percent) |
| --- | --- | --- | --- | --- | --- |
| **bSM** | 40 | 5% | 1h | 0.75 | 0 |
|  |  |  |  | 1.5 | 44.1±10.3 |
|  |  |  |  | 3 | 98.7±0.89 |
|  |  |  | 2h | 0.75 | 0 |
|  |  |  |  | 1.5 | 41.5±8.6 |
|  |  |  |  | 3 | 99.7±0.4 |
|  |  |  | 4h | 0.75 | 0.35±0.07 |
|  |  |  |  | 1.5 | 95.8±3.1 |
|  |  |  |  | 3 | 100 |
| POPC | 40 | 5% | 1h | 0.75 | 0.45±0.3 |
|  |  |  |  | 1.5 | 0.15±0.15 |
|  |  |  |  | 3 | 2.2±0.3 |
|  |  |  | 2h | 0.75 | 0.1±0.16 |
|  |  |  |  | 1.5 | 0.07±0.12 |
|  |  |  |  | 3 | 1.7±1.3 |
|  |  |  | 4h | 0.75 | 1.1±0.12 |
|  |  |  |  | 1.5 | 1.7±0.5 |
|  |  |  |  | 3 | 27±0.8 |
| POPE | 40 | 5% | 1h | 0.75 | 2.3±0.8 |
|  |  |  |  | 1.5 | 20.9±4.2 |
|  |  |  |  | 3 | 22.3±0.1 |
|  |  |  | 2h | 0.75 | 0 |
|  |  |  |  | 1.5 | 11.7±2.1 |
|  |  |  |  | 3 | 10.1±1.7 |
|  |  |  | 4h | 0.75 | 2.4±0.21 |
|  |  |  |  | 1.5 | 15.2±3.8 |
|  |  |  |  | 3 | 17.2±0.2 |
| POPS | 40 | 5% | 1h | 0.75 | 8.9±0.9 |
|  |  |  |  | 1.5 | 2.5±0.7 |
|  |  |  |  | 3 | 0.2±0.1 |
|  |  |  | 2h | 0.75 | 8.3±0.2 |
|  |  |  |  | 1.5 | 2.7±1.1 |
|  |  |  |  | 3 | 0 |
|  |  |  | 4h | 0.75 | 12.5±0.3 |
|  |  |  |  | 1.5 | 6.3±1.0 |
|  |  |  |  | 3 | 1.7±1.1 |
| bSM | 20 | 5% | 1h | 1.5 | 34.0±2.4 |
|  |  |  |  | 0.75 | 20.1±0.8 |
|  |  |  |  | 0.375 | 16.0±1.3 |
|  |  |  |  | 0.187 | 13.9±1.7 |
|  |  |  |  |  |  |
|  |  | 15% |  | 1.5 | 2.6±0.5 |
|  |  |  |  | 0.75 | 2.6±1.3 |
|  |  |  |  | 0.375 | 2.2±1.8 |
|  |  |  |  | 0.187 | 6.7±0.9 |
| POPC | 20 | 5% | 1h | 1.5 | 15.2±2.1 |
|  |  |  |  | 0.75 | 2.6±1.9 |
|  |  |  |  | 0.375 | 0.9±1.3 |
|  |  |  |  |  |  |
|  |  | 15% |  | 1.5 | 4.9±2.2 |
|  |  |  |  | 0.75 | 4.7±1.3 |
|  |  |  |  | 0.375 | 4.8±1.8 |
| POPS | 20 | 5% | 1h | 3 | 4.2±1.4 |
|  |  |  |  | 1.5 | 0 |
|  |  |  |  | 0.75 | 0 |
|  |  |  |  |  |  |
|  |  | 15% |  | 3 | 0.9±0.2 |
|  |  |  |  | 1.5 | 0 |
|  |  |  |  | 0.75 | 0 |
| SM 35:1 | 40 | 5% | 1h | 1.5 | 19.6±3.4 |
|  | 40 | 5% |  | 0.75 | 19.0±2.1 |
|  | 40 | 15% |  | 1.5 | 30.6±4.6 |
|  | 40 | 15% |  | 0.75 | 33.4±1.7 |
|  | 20 | 5% |  | 1.5 | 69.3±5.2 |
|  | 20 | 5% |  | 0.75 | 71.0±3.8 |
|  | 20 | 5% |  | 0.375 | 18.7±2.3 |
|  | 20 | 15% |  | 1.5 | 59.2±4.7 |
|  | 20 | 15% |  | 0.75 | 35.6±2.1 |
|  | 20 | 15% |  | 0.375 | 2.9±1.1 |


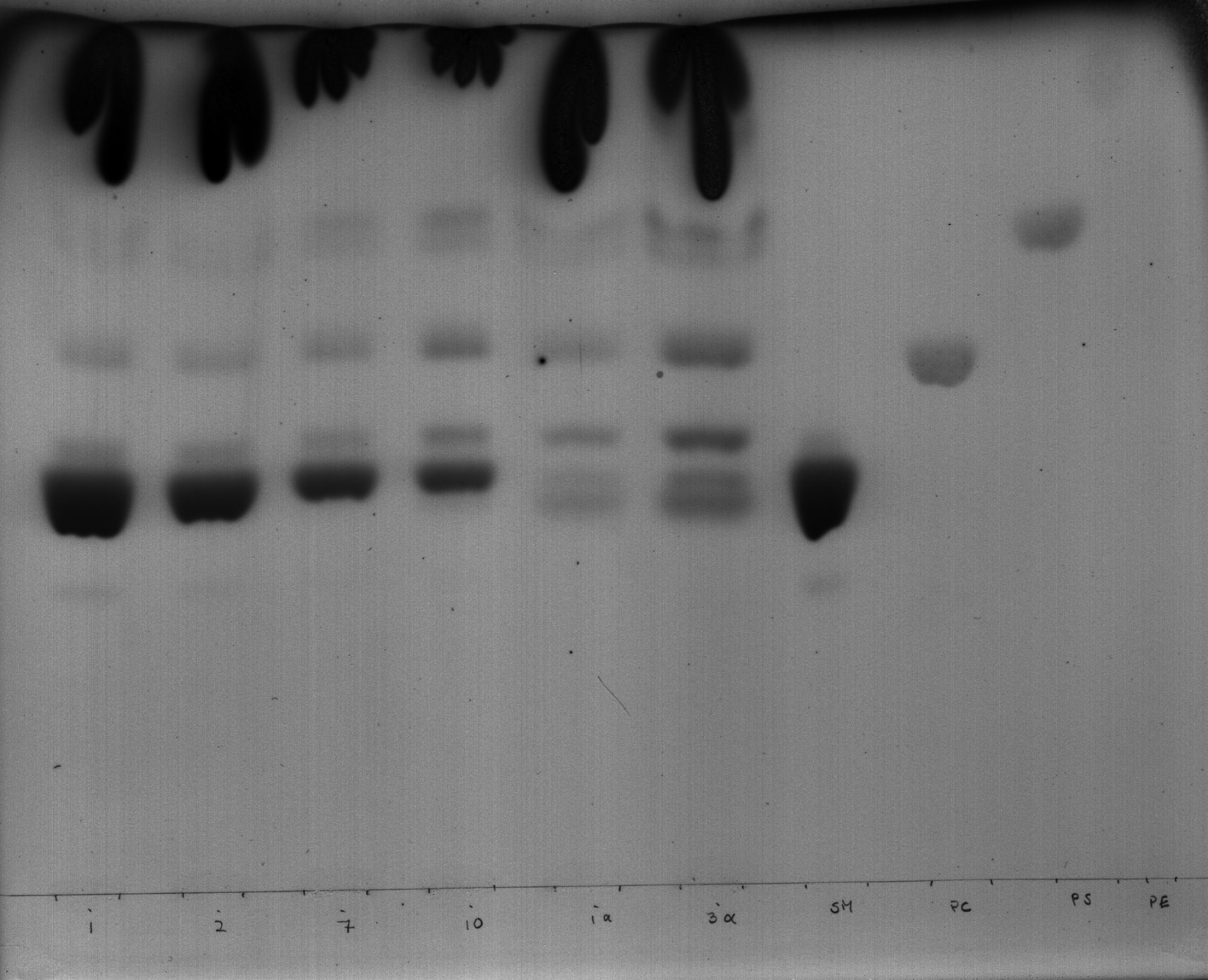


20mM

SM 35:1

0.375mM

20mM

SM 35:1

0.375mM

5%

18.3%

15%

2.5%

SM

PC

PS

Hematocrit content

Hemolysis

MαCD concentration

Loaded lipid

Lipid concentration

Figure S1. Thin layer chromatography of the lipids in the supernatant of the lipid exchange process, after exchange with SM 35:1. The loaded lipid is present in excess in the supernatant, causing the large dark bands in both lanes. The extracted SM is presented as double bands (due to the bimodal fatty acid composition of natural SM); however, SM 35:1 is only covering one of the bands (corresponding to SM species with shorter acyl chains) and one distinct band for the extracted SM can be observed. PS bands can be observed for both lanes, although the hemolysis value for the first lane is small. This shows that exchange with SM 35:1 is not limited to the outer leaflet and some inner leaflet lipids are also extracted. Note that the hematocrit content for the experiments in the two lanes is not the same.


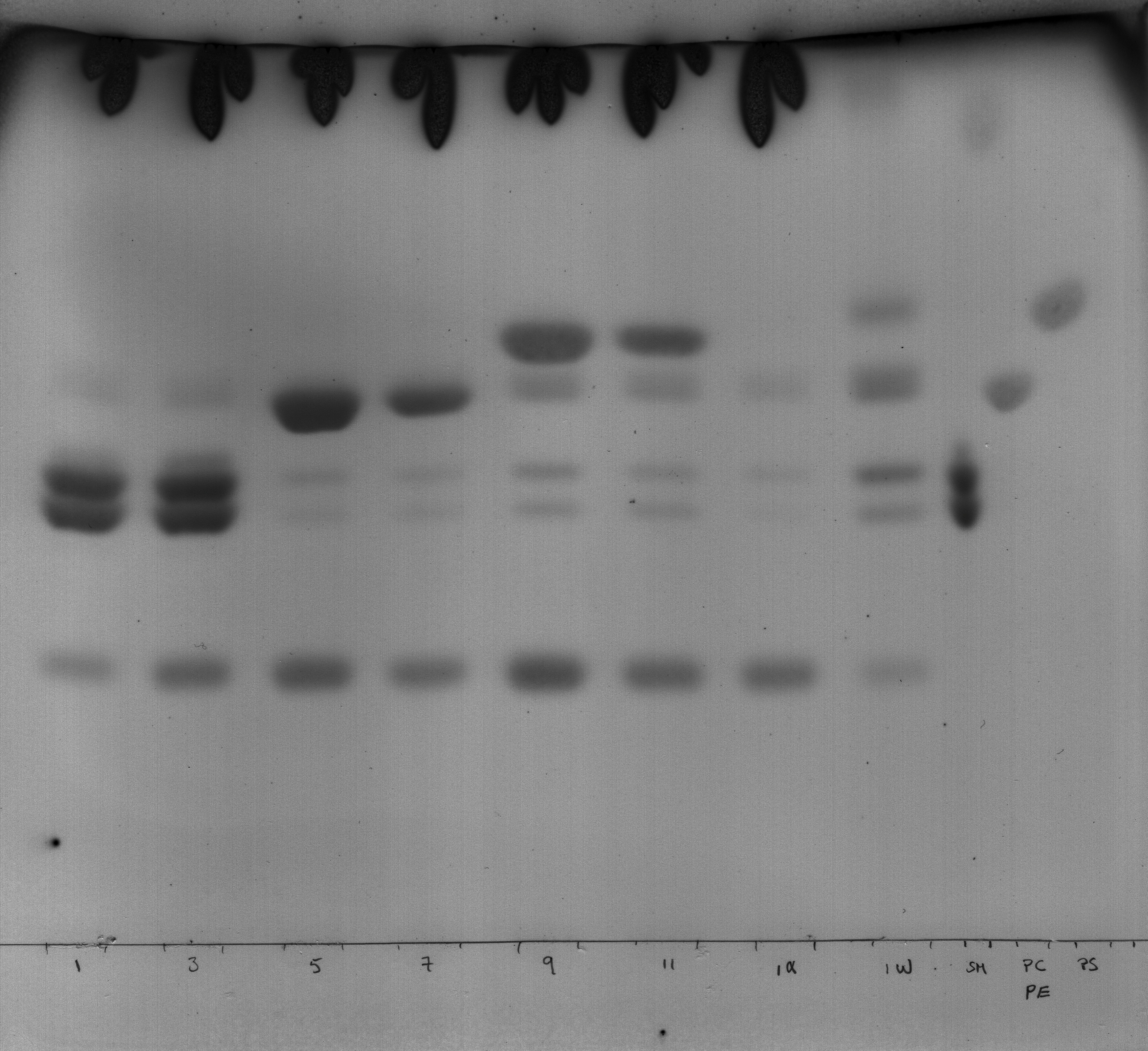


SM

PC

PE

PS

1

3

4

5

6

7

2

8

9

10

11

A

B


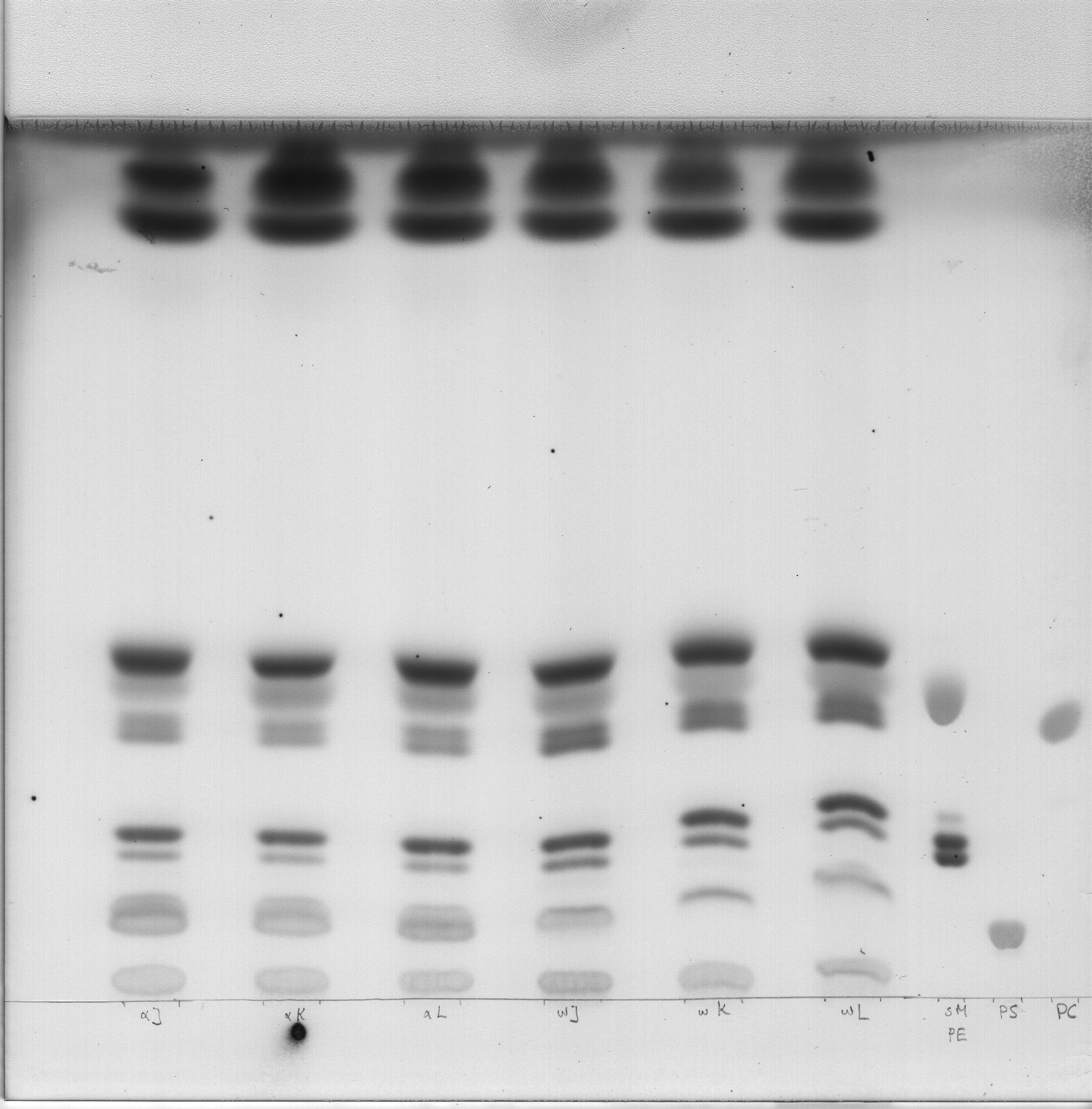


SM

PC

PE

PS

Chol

1

3

4

5

6

2

7

8

9

Figure S2. A) Full length TLC plate of Figure 2A. Note that lane 8 in this image corresponds to lane 7 of Figure 2. Pure lipids are loaded in lanes 9-11 as controls. The dark smeared bands on top of lanes 1-7 is due to the presence of MαCD in the loaded samples. B) Full length TLC plate of Figure 2B. Pure lipids are loaded in lanes 7-9 as controls.


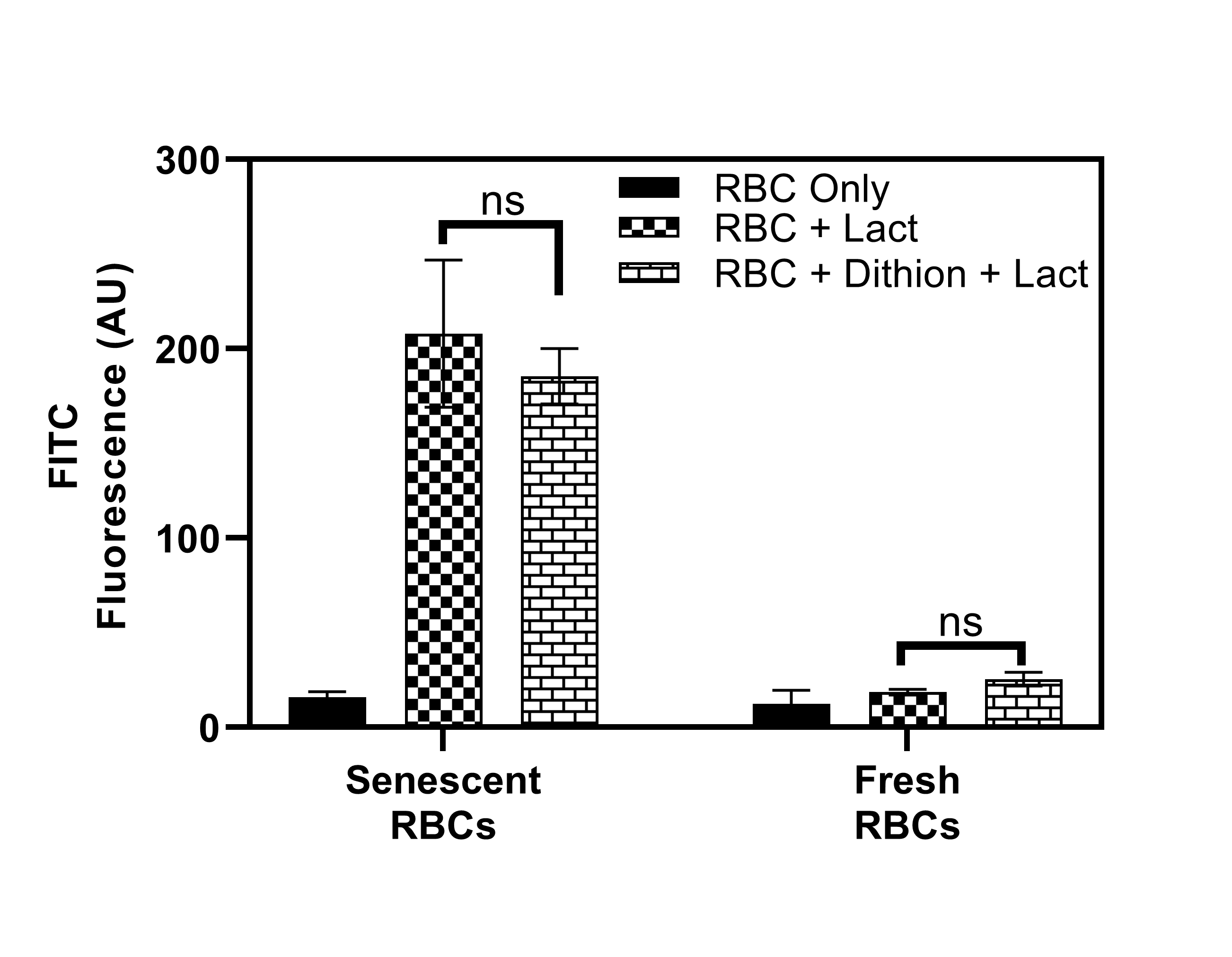


Figure S3. PS detection in the outer leaflet of fresh and expired RBCs before and after treatment with sodium dithionite. The loss of asymmetry and subsequent translocation of PS to the outer leaflet after treatment with sodium dithionte was detected using FITC conjugated lactadherin. Senescent RBCs express PS on their outer leaflet and thus show an intense fluorescence signal when treated with lactadherin-FITC. Treatment of senescent RBCs with sodium dithionite did not significantly change the fluorescence intensity. No significant changes in the fluorescence levels were recorded for fresh RBCs before and after the treatment with sodium dithionte and lactadherin-FITC. Lact and Dithion denote lactadherin-FITC and sodium dithionite, respectively. Statistical analysis was performed using two way ANOVA analysis. NS denotes not significant (p value>0.05).
